# Neutralizing antibodies from the rare convalescent donors elicited antibody-dependent enhancement of SARS-CoV-2 variants infection

**DOI:** 10.3389/fmed.2022.952697

**Published:** 2022-10-19

**Authors:** Song Mu, Shuyi Song, Yanan Hao, Feiyang Luo, Ruixin Wu, Yi Wang, Xiaojian Han, Tingting Li, Chao Hu, Shenglong Li, Meiying Shen, Jingjing Huang, Wang Wang, Yingming Wang, Aishun Jin

**Affiliations:** ^1^Department of Immunology, College of Basic Medicine, Chongqing Medical University, Chongqing, China; ^2^Chongqing Key Laboratory of Basic and Translational Research of Tumor Immunology, Chongqing Medical University, Chongqing, China; ^3^Department of Breast Surgery, The First Affiliated Hospital of Chongqing Medical University, Chongqing, China

**Keywords:** antibody-dependent enhancement, neutralizing antibody, infection-enhancing antibodies, SARS-CoV-2 variants, receptor-binding domain (RBD)

## Abstract

Currently, neutralizing antibody and vaccine strategies have been developed by targeting the SARS-CoV-2 strain identified during the early phase of the pandemic. Early studies showed that the ability of SARS-CoV-2 RBD or NTD antibodies to elicit infection enhancement *in vivo* is still controversial. There are growing concerns that the plasma and neutralizing antibodies from convalescent patients or people receiving vaccines mediate ADE of SARS-CoV-2 variants infections in immune cells. Here, we constructed engineered double-mutant variants containing an RBD mutation and D614G in the spike (S) protein and natural epidemic variants to gain insights into the correlation between the mutations in S proteins and the ADE activities and tested whether convalescent plasma and TOP10 neutralizing antibodies in our laboratory mediated the ADE effects of these SARS-CoV-2 variants. We found that one out of 29 convalescent plasma samples caused the ADE effect of pandemic variant B.1.1.7 and that the ADE effect of wild-type SARS-CoV-2 was not detected for any of these plasma samples. Only one antibody, 55A8, from the same batch of convalescent patients mediated the ADE effects of multiple SARS-CoV-2 variants *in vitro*, including six double-mutant variants and four epidemic variants, suggesting that ADE activities may be closely related to the antibody itself and the SARS-CoV-2 variants' S proteins. Moreover, the ADE activity of 55A8 depended on FcγRII on immune cells, and the introduction of LALA mutations at the Fc end of 55A8 eliminated the ADE effects *in vitro*, indicating that 55A8^LALA^ may be a clinical drug used to prevent SARS-CoV-2 variants. Altogether, ADE may occur in rare convalescent patients or vaccinees with ADE-active antibodies who are then exposed to a SARS-CoV-2 variant. These data suggested that potential neutralizing antibodies may need to undergo ADE screening tests for SARS-CoV-2 variants, which should aid in the future design of effective antibody-based therapies.

## Introduction

As SARS-CoV-2 strains continue to evolve, new variants have been increasing in the current global pandemic. Obviously, the SARS-CoV-2 variants causing the global pandemic are antigenically distinct from the previous prototype during the early phase of the pandemic. Current neutralizing antibodies and vaccine strategies were developed by targeting the prototype SARS-CoV-2 strain identified during the early phase of the pandemic (1–6). We also reported the isolation and characterization of several hundreds of RBD-specific mAbs from SARS-CoV-2-infected individuals (7), some of which could neutralize authentic SARS-CoV-2 variants (8). Previous studies have shown that some RBD or NTD antibodies mediate antibody-dependent enhancement (ADE) for wild-type SARS-CoV-2 *in vitro*, but that is still controversial *in vivo* (9, 10). Thus, a safety concern for the clinical use of neutralizing antibodies or plasma from convalescent patients is the ADE of these SARS-CoV-2 variant infections.

Antibody-dependent enhancement has been observed for coronaviruses, and it is often mediated by Fc receptors (FcRs) on different immune cells for immunoglobulin G (IgG) (11, 12). In SARS-CoV infection, studies have demonstrated FcR-IgG-mediated ADE in ACE2-negative cells (11, 13, 14). A novel mechanism for ADE in MERS-CoV has demonstrated that both the Fc and Fab portions of anti-MERS mAb are required for antibody-mediated viral entry, suggesting that the Fab-Spike complex was associated with ADE activity (15). Multiple studies have reported FcR-independent infection enhancement of wild-type SARS-CoV-2 *in vitro* but have not exhibited infection enhancement in animal model experiments (9, 10). Obviously, the antigenically of spike (S) protein on the surface of the worldwide SARS-CoV-2 variants differs from the previous wild type. Therefore, the ability of neutralizing antibodies to mediate the enhancement of new SARS-CoV-2 variant infection is unknown, but is a theoretical concern for COVID-19 antibody-based therapies development.

As SARS-CoV-2 strains continue to evolve, SARS-CoV-2 variants are replacing formerly dominant strains and sparking new COVID-19 epidemics (16), i.e., B.1.1.7 (broke out in the United Kingdom), B.1.351 (broke out in South Africa), and B.1.1.28 (broke out in Brazil). Most of these variants contain the D614G mutation and mutations of the receptor-binding domain (RBD) in the spike protein. Several studies have established that multiple of these mutations could increase the transmissibility of SARS-CoV-2 in ACE2-positive cells (17, 18). However, it has not been fully documented whether neutralizing antibodies or plasma from convalescent patients mediates ADE of these SARS-CoV-2 variant infections in immune cells. Therefore, we constructed engineered double-mutant variants containing an RBD mutation and D614G in the spike protein and natural epidemic variants to gain insights into the correlation between the mutations in the spike protein and the ADE of SARS-CoV-2 variant infection.

Here, we constructed a series of SARS-CoV-2 variants, including engineered double-mutant variants containing an RBD mutation and D614G in the spike protein and natural epidemic variants, and tested whether plasma samples and TOP10 neutralizing antibodies in our laboratory from convalescent patients mediated the ADE effects of these SARS-CoV-2 variants. We found that one out of 29 convalescent plasma samples caused the ADE effect, and one potential neutralizing antibody, 55A8, from the same batch of convalescent patients mediated the ADE effects for most of the SARS-CoV-2 variants *in vitro*. Furthermore, the ADE activity of 55A8 depended on FcγRII on immune cells, and the introduction of LALA mutations at the Fc end of 55A8 eliminated the ADE effects. The study demonstrated that the neutralizing antibodies from convalescent patients mediated the ADE of SARS-CoV-2 variant infection *in vitro*, providing additional evidence to better understand the biology of new SARS-CoV-2 variants for current antibody therapies and vaccine protection.

## Materials and methods

### Cell lines

HEK 293T cells, Daudi cells, Raji cells, and K562 cells were purchased from the American Type Culture Collection (ATCC, Manassas, VA, USA). HEK 293T cells were maintained in Dulbecco's modified Eagle's medium (DMEM; Gibco™, USA) supplemented with 10% fetal bovine serum (FBS; Gibco, Rockville, MD, USA), 100 mg/ml of streptomycin, and 100 units/ml of penicillin at 37°C in 5% CO2. HEK 293T cells transfected with human ACE2 (293T-ACE2) were cultured under the same conditions (7). Daudi, Raji, and K562 cell lines were cultured at 37 °C in Roswell Park Memorial Institute 1640 Medium (RPMI 1640 medium; Gibco™, USA) with 10% FBS.

### Convalescent patients' plasma

Human plasma samples from 29 COVID-19 convalescent patients in Chongqing Medical University Affiliated Yongchuan Hospital were collected on 15 March 2020 and 16 March 2020 (7). All volunteers signed informed consent forms.

### Plasmid construction and antibody expression

The pMD2.G plasmid encoding the wild-type SARS-CoV-2 spike (S) gene was generated as previously described^7^. The D614G plasmid and the double-mutant variant plasmids encoding the S gene were constructed by a Phusion site-directed mutagenesis kit (Thermo Scientific, USA), with the wild-type S gene plasmid as a template. Following the procedure of the mutagenesis kit to amplify variants by circular PCR, 15 to 20 nucleotides before and after the target mutation site were selected as forward primers, while the reverse complementary sequences were selected as reverse primers. Following site-directed mutagenesis PCR, the template chain was digested using *Dpn I* restriction endonuclease (NEB, USA). Afterward, the PCR product was directly used to transform *E. coli* Stbl3 competent cells; single clones were selected and then sequenced. The frequency of different variants in the epidemic population is shown in Supplementary Table 1, and the primers designed for the spike mutation sites are shown in Supplementary Table 3. The codon-optimized S gene variants encoding the newly epidemic SARS-CoV-2 were synthesized and cloned into pMD2.G vector by Tsingke Biotechnology (Beijing, China), including B.1.1.7, B.1.351, B.1.1.28, B.1.617, B.1.617.2, B.1.1.529.1, and B.1.1.529.2. The SARS-CoV-2 genome and lineage data were downloaded from the BIGD (https://bigd.big.ac.cn/ncov/) and GISAID (https://www.epicov.org/) databases with sample collection date and location information (19–21).

The neutralizing antibodies were generated from SARS-CoV-2 RBD-specific memory B cells using a single B-cell isolation and cloning strategy (7). The heavy chain and light chain plasmids were transiently co-transfected into HEK293 cells followed by purification with Protein A resin. The antibody 55A8^LALA^ was generated by introducing the LALA mutation (L234A and L235A) in the Fc region of IgG1 to abolish binding with FcγRs and prepared using the same protocol used for the generation of wild-type Ab.

### Production of SARS-CoV-2 pseudoviruses

SARS-CoV-2 pseudotyped viruses were produced as previously described (7). Briefly, 1 × 10^6^ HEK 293T cells were cotransfected with 3.8 μg psPAX2, 3.8 μg pWPXL luciferase, and 0.3 μg pMD2. G plasmid encoding SARS-CoV-2 S and mutations of S using Xfect Transfection Reagent (Takara, Japan) according to the manufacturer's instructions on 6-well-plates. The S and mutant S protein pseudotyped viruses in the supernatants were harvested 48 h after transfection, centrifuged at 300 g for 10 min, filtered through a 0.45 μm filter, and stored at −80°C. The titers of the pseudoviruses were calculated by determining the number of viral RNA genomes per ml of viral stock solution using a Lenti-X qRT-PCR Titration Kit (Takara, Japan).

### ADE assays of pseudotyped SARS-CoV-2 infection

Antibody-dependent enhancement assays were performed using the Daudi, Raji, and K562 cell lines. Then, 100 μl of Daudi, Raji, and K562 cells at a density of 2 × 10^4^ cells/ml were seeded 48 h before infection in a 96-well cell culture plate (NEST) coated with 0.01% poly-L-lysine in PBS^22, 23^. Ten microliters of 4-fold serially diluted mAbs or 2-fold serially diluted convalescent plasma were mixed with 40 μl of supernatant containing 2 × 10^6^ copies/μl of pseudovirus. The mixture was incubated for 1 h at 37°C and supplied with 5% CO_2_. Then, the medium was replaced with 50 μl of fresh medium, and the cells were coincubated with mixtures of pseudoviruses and mAbs for 12 h. The mAb concentrations ranged from 0.12 to 8,000 ng/ml. Subsequently, 100 μl of supplemented fresh medium was added to each well for an additional 48 h of incubation. The relative luminescence units (RLU) were measured using luciferase assay reagent (Promega, Madison, WI, USA) according to the manufacturer's protocol. To perform the ADE blocking experiment with the neutralizing antibody 55A8, Daudi cells were blocked with a fresh medium containing 4 μg/ml of purified mouse anti-human CD32 (Cat: 555447, BD Pharmingen) at 37°C for 1 h, and the other operating steps were in accordance with the ADE assay experiments.

### Neutralization assay

Pseudoviruses were generated as previously described. The 50 μl serially diluted antibodies were incubated with pseudovirus (2.0 × 10^6^ copies/μl, 50 μl) at 37°C for 1 h. The mixture of viruses and purified antibodies was then added to a hACE2-expressing cell line (hACE2-293T cells). After 72 h of culture, the luciferase activity of infected hACE2/293T cells was measured by the Bright-Luciferase Reporter Assay System (Promega). The relative luminescence unit (RLU) of Luc activity was detected using the Thermo Fisher LUX reader. Half-maximal inhibitory concentrations (IC_50_) were calculated using four-parameter logistic regression in GraphPad Prism 8.0.

### Statistical analysis

Statistical analysis was carried out using GraphPad Prism 8.0. Data are shown as the mean ± SEM. Two-group comparisons were performed by Student's *t*-test. All tests were two-tailed, and *P* < 0.05 was considered statistically significant.

## Results

### The plasma from convalescent patients against wild-type SARS-CoV-2 mediated the ADE effects of the epidemic variants *in vitro*

**Figure 1:**
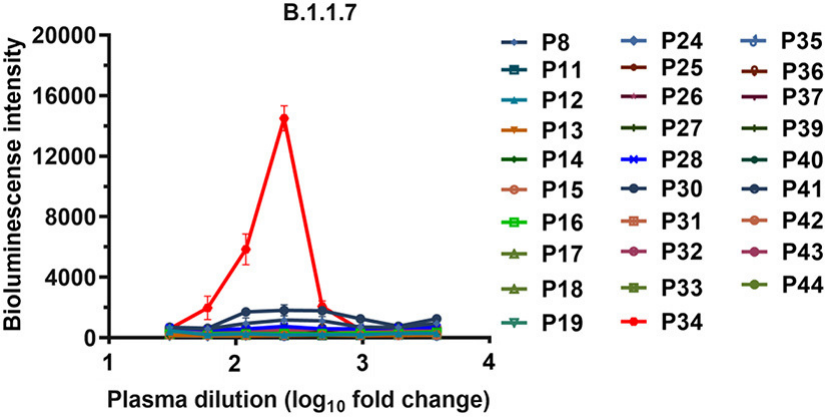
ADE activities of the plasma samples from the COVID-19 convalescent patients. The B.1.1.7 pseudovirus was preincubated with serially diluted plasma samples, and the mixtures were added to Daudi cells to evaluate their ability to enhance infection. Each curve represents an individual plasma sample. RLU values resulting from infection with variant pseudotyped viruses were quantified by a luminescence meter. Data for each plasma sample were obtained from a representative infectivity experiment of three replicates and presented as the mean values ± SEM.

To assess whether the neutralizing antibodies obtained from the convalescent patients mediated the ADE effects of SARS-CoV-2, we first tested the enhancement of SARS-CoV-2 infection for the 29 convalescent plasma samples, which were from our previous research that screened neutralizing antibodies from the convalescent patients of COVID-19 (7). The ADE activities of these convalescent plasma samples were preliminarily screened and confirmed with three concentration dilutions using a magnetic chemiluminescence enzyme immunoassay (MCLIA) and a pseudovirus-based assay by the Wuhan-1 strain (GenBank: MN_908947, as a wild-type strain) and the variant B.1.1.7. Among the 29 convalescent plasma samples, none of the three dilutions mediated the ADE effects of wild-type SARS-CoV-2 (Supplementary Figure 1). However, the P34 plasma sample showed an enhancement for pseudotyped SARS-CoV-2 B.1.1.7 infection, which was indicated by the increase in luciferase expression in Daudi cells (Supplementary Figures 2, 3). We further detected the enhancement of the ADE effects for these 29 plasma samples at serial dilutions by the B.1.1.7. As shown in Figure 1, only the P34 sample showed a concentration-dependent enhancement of infection in Daudi cells, indicating that a small amount of convalescent plasma from convalescent patients mediated the ADE effects of the SARS-CoV-2 variants. Therefore, we speculated that a small number of neutralizing antibodies obtained from convalescent patients with COVID-19 may have ADE activities against SARS-CoV-2 variants.

### Identification of neutralizing antibodies mediating the enhancement of SARS-CoV-2 variant infection

**Figure 2:**
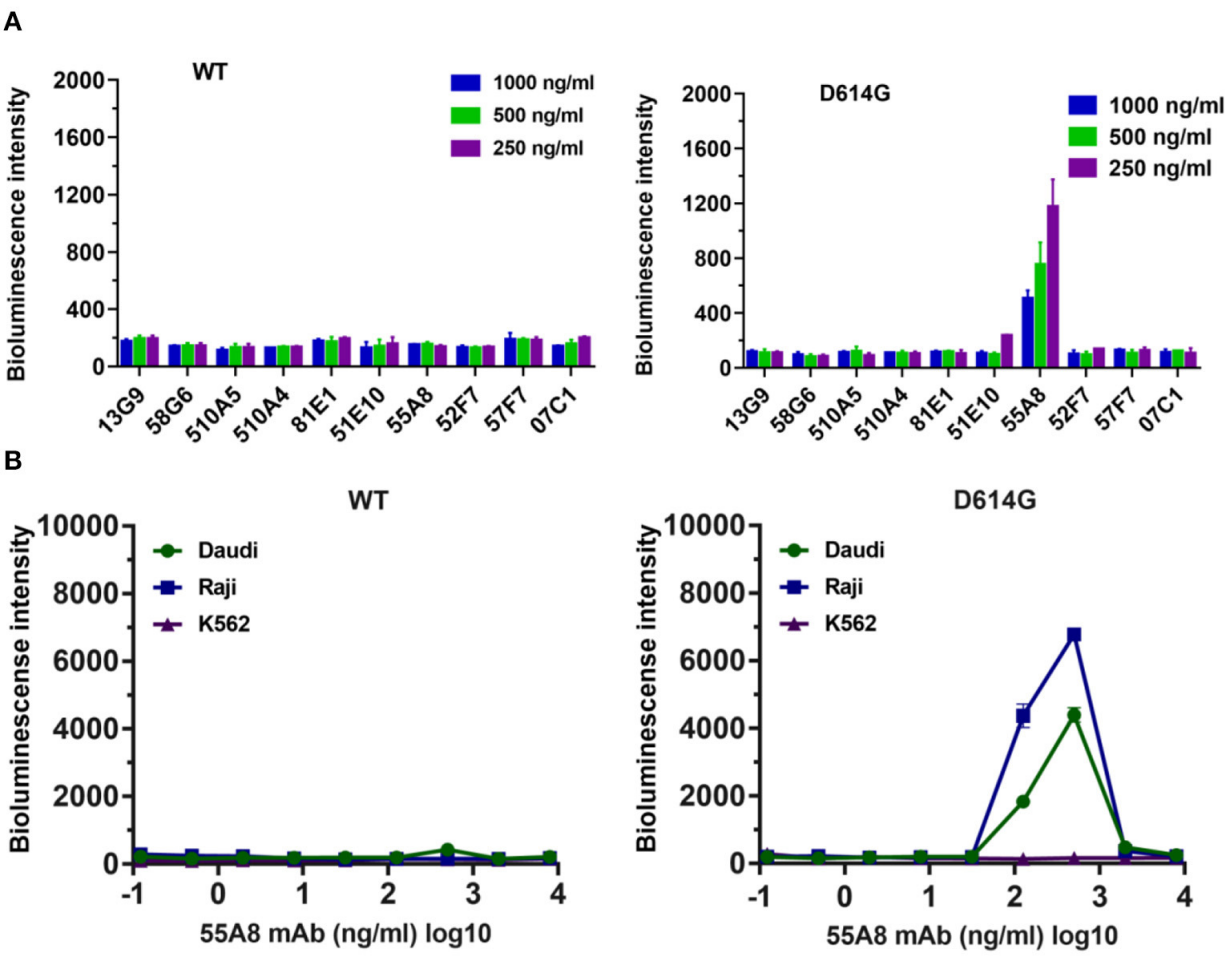
Identification of the neutralizing antibodies (Nabs)-mediated enhancement of SARS-CoV-2 variant infection. **(A)** Assessment of the ADE activities of 10 potent NAbs against SARS-CoV-2 WT and the D614G variant. Pseudoviruses were preincubated with 250, 500, and 1,000 ng/ml of NAbs, and these mixtures were added to Daudi cells to evaluate their ability to enhance infection. **(B)** Assessment of ADE activities of the neutralizing antibody 55A8 for SARS-CoV-2 WT and the D614G variant. Pseudoviruses preincubated with serial dilutions of 55A8 mixtures were added to Daudi, Raji, and K562 cells to evaluate their ability to enhance infection. RLU values resulting from infection with variant pseudotyped viruses were quantified by a luminescence meter. Data for each NAb were obtained from a representative infectivity experiment of three replicates and presented as the mean values ± SEM.

To investigate the ADE activities of the neutralizing antibodies for the SARS-CoV-2 variants, we analyzed mutants of the SARS-CoV-2 spike protein that determine the infectivity of the virus and its transmissibility in the host based on published genomic data (19). The analyses of variants showed that 81.79% of these variants contained the D614G mutant in the spike protein reported in the database in September 2020 (Supplementary Table 1). Therefore, using the codon-optimized S gene of wild-type SARS-CoV-2 (Wuhan-1 strain) as a template, we first constructed the D614G pseudotyped plasmid by site-directed mutagenesis, and the top 10 potential antibodies against D614G from our lab retained neutralizing activities identical or similar to those against wild-type SARS-CoV-2 (Supplementary Figure 4) (22). Next, we evaluated the enhancement of the D614G pseudotyped variant infection for the top 10 potential neutralizing antibodies from our lab by using Daudi cells (7). The results showed that only the neutralizing antibody 55A8 enhanced D614G variant infection at all three concentrations (Figure 2A). Moreover, the ADE activity of 55A8 showed a concentration-dependent enhancement for the D614G pseudotyped variant infection in Daudi cells and Raji cells (Figure 2B), suggesting that the neutralizing antibody 55A8 may be an ADE antibody of SARS-CoV-2 variants. According to our previous antibodies screening records, the neutralizing antibody 55A8 gene was isolated from a mixed memory B-cell sample containing the P8, P11, P12, P17, P18, P30, P31, P34, and P35 samples (7). Although the P34 plasma sample showed an ADE activity, it was difficult to determine which patient the antibody 55A8 originated from as the neutralizing antibodies derived from memory B cells may be different from those obtained from the convalescent plasma. Additionally, we further analyzed the usage of antibody variable-gene segments for variable (V) genes (Supplementary Table 2). We found that the heavy chain of 55A8 was encoded by IGHV1-69, and the IGHV1-69 gene could pair with the light chain V gene IGKV1-5 (Supplementary Table 2), which was consistent with some ADE antibodies previously reported (23, 24). In summary, the neutralizing antibody 55A8 obtained from convalescent patients against wild-type SARS-CoV-2 mediated the ADE effects of the D614G variant.

### The neutralizing antibody 55A8 mediated antibody-dependent enhancement of SARS-CoV-2 variant infection

As previously described, the early database showed that most of the RBD (neutralizing antibody-binding domain) mutants reported in September 2020 were found with the D614G mutant (Supplementary Table 1). We next selected two important mutants containing an RBD mutant and the D614G mutant to construct double-mutant pseudotyped viruses to confirm the ADE of SARS-CoV-2 variant infection mediated by 55A8. Therefore, 49 double-mutant variants were successfully constructed (Supplementary Figure 5). Of all 49 pseudotyped variants, only seven were determined to have high infectivity, and the RLU levels were 1.5-fold higher than those of the D614G strain, including D614G+P330S, D614G+F338L, D614G+A348T, D614G+N439K, D614G+G446V, D614G+T478I, and D614G+H519Q (Supplementary Figure 5). Among them, the results confirmed that the multiple mutations changed the infectivity of the SARS-CoV-2 variants, e.g., D614G, N439K, G446V, and H519Q, which was consistent with previous reports (25–28). Interestingly, the top 10 potential antibodies from our lab retained good neutralizing activities against these seven double-mutant SARS-CoV-2 variants (Supplementary Figure 5).

**Figure 3:**
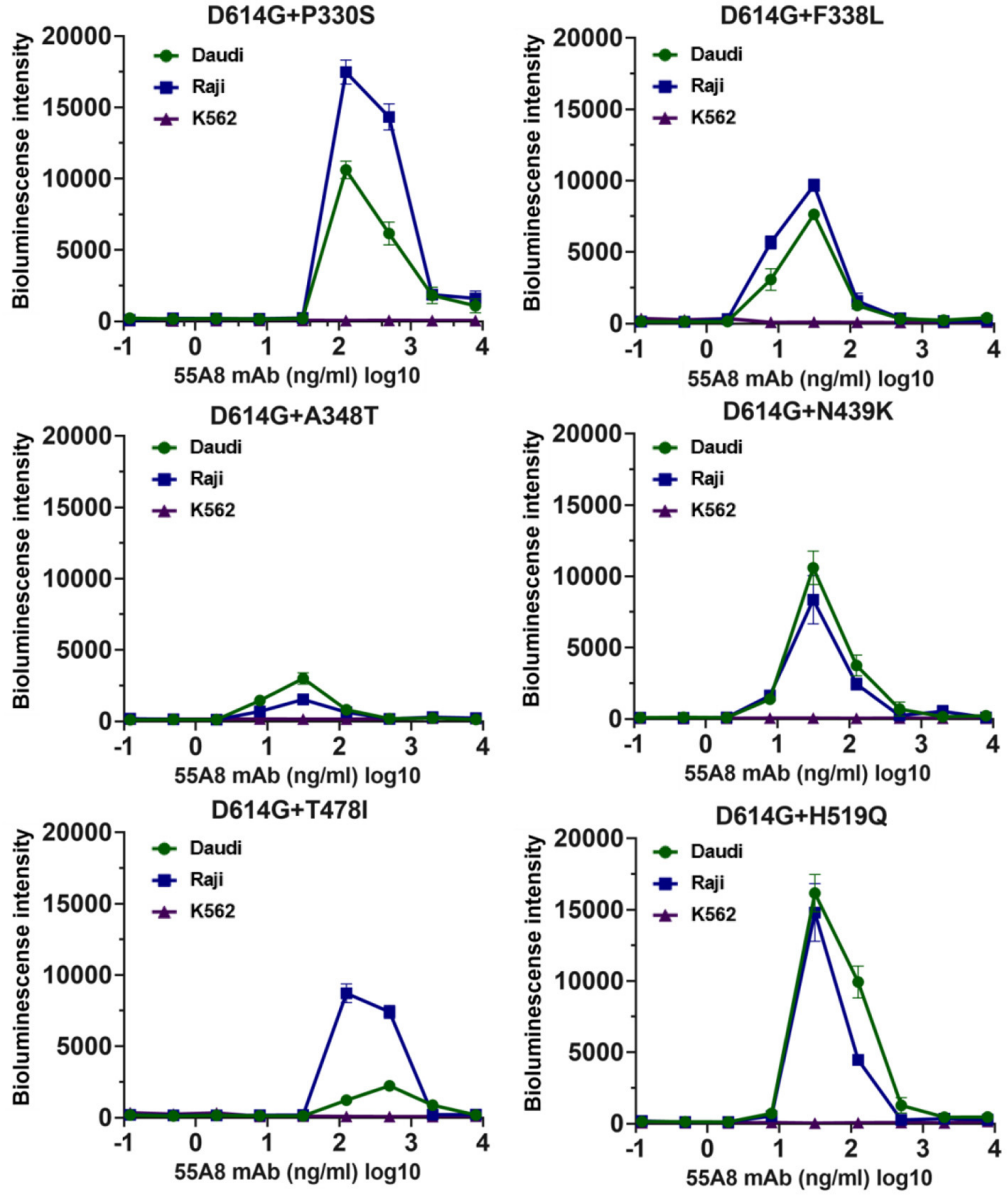
ADE activities of the neutralizing antibody 55A8 for the high-infective double-mutant variants. The highly infectious double-mutant variants included D614G+P330S, D614G+F338L, D614G+A348T, D614G+N439K, D614G+T478I, and D614G+H519Q. Pseudoviruses were preincubated with serial dilutions of 55A8 mAbs, and these mixtures were added to Daudi, Raji, and K562 cells to evaluate their ability to enhance infection. RLU values resulting from infection with variant pseudotyped viruses were quantified by a luminescence meter. Data for each NAb were obtained from a representative infectivity experiment of three replicates and presented as the mean values ± SEM.

Then, we evaluated the enhancement of the seven highly infectious double-mutant variant infections for the top 10 potential neutralizing antibodies from our lab at three antibody concentrations by using Daudi cells. The results also showed that only the potential neutralizing antibody 55A8 showed an enhancement of the six high-infectivity double-mutant variant infections (including D614G+P330S, D614G+F338L, D614G+A348T, D614G+N439K, D614G+T478I, and D614G+H519Q), except SARS-CoV-2 D614G+G446V, as indicated by the increase in luciferase expression in Daudi cells (Supplementary Figure 6). We further detected the enhancement of the double-mutant variant infection for 55A8 at serial dilutions in Daudi, Raji, and K562 cells. The neutralizing antibody 55A8 showed a concentration-dependent enhancement of infection at six SARS-CoV-2 variants in Daudi cells and Raji cells (Figure 3), but the ADE effect of D614G+G446V was not observed (Supplementary Figure 7). The antibody concentration range of the 55A8 ADE activity was from 7.81 ng/ml to 2000.00 ng/ml, and the highest ADE levels of these variants containing different RBD mutation sites were different, suggesting that these mutations of RBD may be related to these variants' ADE activities. Taken together, these results confirmed that the neutralizing antibody 55A8 was an ADE antibody.

**Figure 4:**
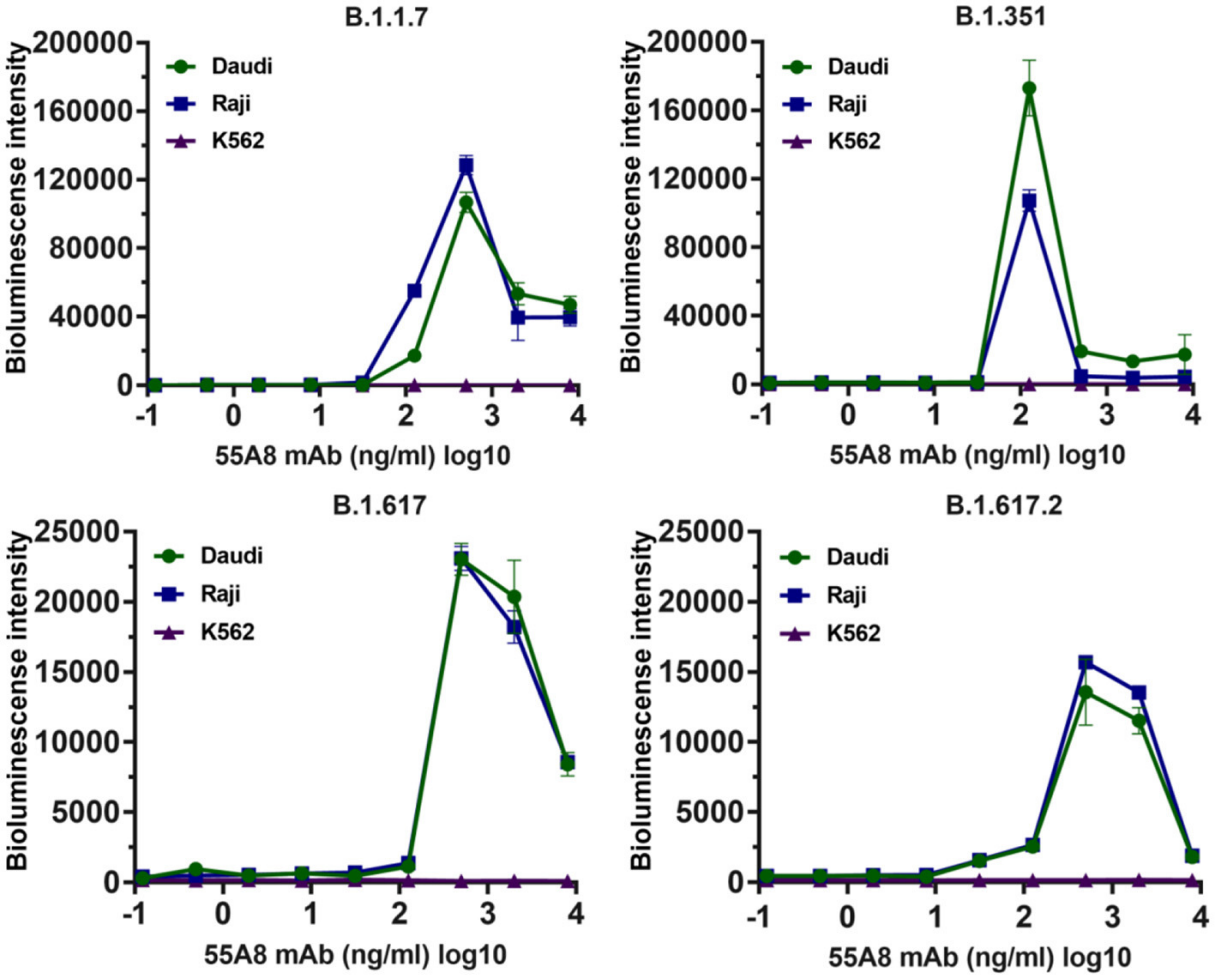
The ADE activities of 55A8 for the newly emerged variants. New epidemic SARS-CoV-2 variants (including B.1.1.7, B.1.351, B.1.617, and B.1.617.2) were preincubated with serially 55A8, and their mixtures were added to Daudi, Raji, and K562 cells to evaluate their ability to enhance infection. RLU values resulting from infection with variant pseudotyped viruses were quantified by a luminescence meter. Data for each NAb were obtained from a representative infectivity experiment of three replicates and presented as the mean values ± SEM.

Additionally, studies on ADE effects using 55A8-mediated new epidemic SARS-CoV-2 variants (including B.1.1.7, B.1.351, B.1.1.28, B.1.617, B.1.617.2, B.1.1.529.1, and B.1.1.529.2) were also evaluated. As shown in Figure 4, the assay results indicated that the diluted neutralizing antibody 55A8 mediated the ADE effects of four epidemic SARS-CoV-2 variants (including B.1.1.7, B.1.351, B.1.617, and B.1.617.2), but the ADE effect of the B.1.1.28, B.1.1.529.1, and B.1.1.529.2 strains was not observed (Supplementary Figure 7). Therefore, these results have shown that the neutralizing antibody 55A8 can mediate the ADE effects of most double-mutant variants and the epidemic variants, indicating that ADE activity may be closely related to the antibody characteristics.

### The neutralizing antibody 55A8 caused ADE effects depending on the Fc receptors

**Figure 5:**
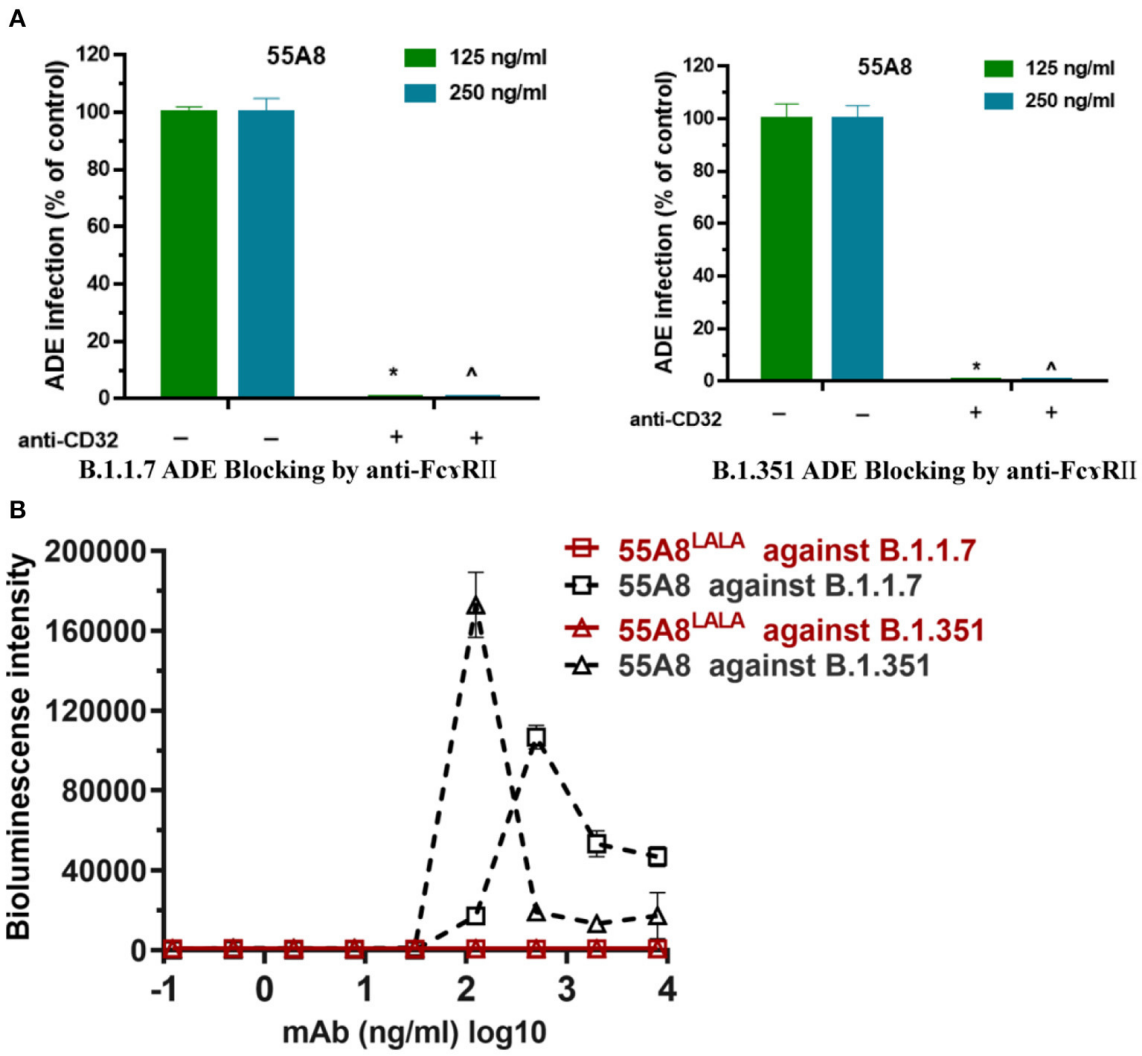
The ADE effects mediated by the neutralizing antibody 55A8 depend on FcγRII. **(A)** Assessment of ADE activities of the neutralizing antibody 55A8 for the epidemic variants (including B.1.1.7 and B.1.351) with or without anti-CD32 (blocking FcɤR II). The SARS-CoV-2 B.1.1.7 and B.1.351 pseudoviruses were preincubated with 125 ng/ml and 250 ng/ml of 55A8, and their mixtures were added to Daudi cells to evaluate their ability to enhance infection. The percentage of control-represented ADE infection was quantified by luminescence meter and normalized to the reference groups without anti-CD32 produced in parallel, and the anti-CD32 was diluted to 40 ng/ml. **(B)** Assessment of ADE activities of 55A8^LALA^ for epidemic variants, including B.1.1.7 and B.1.351. Pseudoviruses were preincubated with serially diluted 55A8 or 55A8^LALA^, and their mixtures were added to Daudi cells to evaluate their ability to enhance infection. RLU values resulting from infection with variant pseudotyped viruses were quantified by a luminescence meter. Data for each NAb were obtained from a representative infectivity experiment of three replicates, presented as the mean values ± SEM, and *p*-values were calculated via two-sided Student's *t*-test. *p < 0.05 vs. the 125 ng/ml of 55A8 group without blocking anti-FcγRII antibody, and ^∧^*p* < 0.05 vs. the 500 ng/ml of 55A8 group without blocking anti-FcγRII antibody.

Fc receptors (FcRs) have been shown to enhance antibody-dependent infectivity in several viral infections, including dengue fever virus, severe acute respiratory syndrome (SARS), and Middle East respiratory syndrome (MERS) coronavirus infections (11, 29, 30). Recent studies have shown that the ADE antibodies of SARS-CoV-2 have at least two mechanisms (9, 31): RBD-specific antibodies depend on Fc-FcγRII, while NTD-specific antibodies depend on changing the conformation of the S protein, which affects the binding of the S protein to the receptor ACE2. Therefore, the RBD-specific antibody 55A8 was used to confirm whether the enhancements of these new SARS-CoV-2 variant infections were mediated by Fc-FcγRII. In this study, we used anti-CD32 (blocking FcγRII) to block the cell surface FcγR receptor to evaluate the engagement of the FcγR receptor in promoting SARS-CoV-2 variants infection. As shown in Figure 5A, the addition of the blocking anti-FcγRII antibody eliminated the enhancement of B.1.1.7 and B.1.351 infection by the neutralizing antibody 55A8, which is similar to the ADE of coronavirus infections, including SARS-CoV, MERS-CoV, Zika, and dengue viruses (32). Additionally, previous studies have shown that the introduction of a LALA mutation at the Fc end of the antibody can eliminate the ADE effects without decreasing its neutralizing activity (9). We also introduced the LALA mutation to the Fc region of 55A8 (55A8^LALA^) to decrease the engagement of 55A8 with FcγRs. Interestingly, no ADE activities were detected for 55A8^LALA^ on all two new SARS-CoV-2 variants, including B.1.1.7 and B.1.351 (Figure 5B). Therefore, these results showed that the ADE activity mediated by 55A8 was dependent on FcγRII expressed in immune cells, and the neutralizing antibody 55A8^LALA^ could still be used to prevent SARS-CoV-2 variants.

## Discussion

The SARS-CoV-2 variants are of concern because of their rapid increase to dominance as well as their unusually large number of mutations in the spike protein, which could lead to changes in mAb therapies and vaccine protection. There are growing concerns that the ADE of SARS-CoV-2 variant infection may affect the safety of therapeutic Abs agents and vaccines. In this study, we described the ADE of SARS-CoV-2 variant infection mediated by plasma samples and neutralizing antibodies obtained from convalescent patients.

Currently, strategies for neutralizing antibodies and vaccines have been developed by targeting the prototype SARS-CoV-2 Wuhan-Hu-1 strain. Notably, the new SARS-CoV-2 variants circulating around the world are antigenically distinct from the previous type. For example, the ongoing Omicron variants have been increasing in the current global pandemic. In this study, we constructed two types of SARS-CoV-2 variants, including engineered double-mutant variants containing an RBD mutation and D614G in the spike protein and natural epidemic variants, to assess the ADE activities for the convalescent plasma samples and neutralizing antibodies. Interestingly, we found that one out of 29 convalescent plasma samples caused the ADE effects of pandemic variant B.1.1.7, suggesting that the rare convalescent plasma from convalescent patients mediated the ADE effects of the SARS-CoV-2 variants. Moreover, we also detected the enhancement of B.1.1.7 infection for the 17 vaccinees' plasma samples. Notably, among the 17 vaccinee**s'** plasma samples, the results showed that one vaccinee plasma sample showed an enhancement for pseudotyped B.1.1.7 infection (Supplementary Figure 8). Thus, these results showed that the rare ADE activities can be observed *in vitro* in the convalescents and vaccinee**s**, indicating that the neutralizing antibodies obtained from convalescent patients or vaccinee**s** may have ADE activities against SARS-CoV-2 variants.

Then, our further study found that only one potent neutralizing antibody obtained from convalescent patients, 55A8, triggered the ADE effects for most of the engineered double-mutant variants except D614G+G446V, and it also mediated the ADE of four of seven new epidemic SARS-CoV-2 variant infections, including B.1.1.7, B.1.351, B.1.617, and B.1.617.2, which was consistent with previously described engineered double-mutant variants. These results indicated that the ADE effects may be closely related to the characteristics of the antibody itself. In addition, the antibody 55A8 retained neutralizing activities against Omicron strains (B.1.1.529.1 and B.1.1.529.2)^34^, and a previous study reported that the Omicron variants (B.1.1.529.1 and B.1.1.529.2), B.1.1.7 and B.1.351 contained multiple shared key mutation sites in the SARS-CoV-2 S protein (33–35). Thus, it is necessary to clarify whether the antibody 55A8 mediates the ADE effects of the Omicron strains. Interestingly, we found that 55A8 was unable to mediate the ADE effects of Omicron variants. Therefore, the relationship between the ADE effects mediated by the 55A8 and the shared key mutations of spike was not clear, and we speculate that it may be related to the binding conformations of this antibody and these S proteins.

In our previous study (22), we performed epitope mapping for the top 20 neutralizing antibodies (including 55A8) in our laboratory via competitive ELISA. The results showed that 16 out of these 20 neutralizing antibodies were grouped into the epitopes recognized by a neutralizing antibody 13G9 (13G9e), but the neutralizing antibody 55A8 had no competition with 13G9. Structural analysis revealed that 13G9 recognizes the steric region S^470 − 495^ in the wild-type RBD^24^, while 55A8 recognizes S^345 − 352^ and S^440 − 450^ in the Omicron RBD^34^. The previous study reported that peptides from the S1 region, including S^304 − 323^, S^364 − 383^, S^364 − 403^, S^454 − 473^, S^484 − 503^,S^544 − 563^, S^564 − 583^, and S^574 − 593^, dramatically blocked the ADE of patient plasma by peptide scanning (36), but these epitopes don't contain the antigen mutation epitopes recognized by the ADE-inducible antibody 55A8. Therefore, these results suggested that some new antigen mutation epitopes recognized by the antibody 55A8 may be associated with ADE activities.

Moreover, the ADE activities analysis of multiple nAbs with clear epitope information, such as 55A8, 58G6, and 13G9, were completed in this study. Barnes et al. (37) classified the neutralizing antibodies (NAbs) targeting the RBD into four classes (classes 1–4) according to their neutralizing mechanisms. The previous study reported that the ADE-inducible neutralizing antibody 7F3 (class 2 NAb) bound to the spike proteins with one “up” and two “down” RBD domains (36), while the antibody 55A8 (class 3 NAb) has no ADE activity when it against the Omicron variants in this study, which also contained the RBD accessibility epitopes in “up/down” conformations (38). This study also revealed that the neutralizing antibodies 58G6 (class 1 NAb) and 13G9 (class 1 NAb) are bound to the spike protein with three “up” RBD domains (22), which have no ADE activities. Therefore, we speculated that there were other factors that influenced ADE activities in addition to RBD accessibility epitopes in the “up/down” conformation, and the specific mechanisms still need to be further studied.

Previous studies have shown that ADE antibodies against SARS-CoV-2 have at least two mechanisms (9, 31): RBD-specific ADE antibodies rely on Fc-FcγRII, while NTD-specific ADE antibodies affect the binding of S proteins to the receptor ACE2, altering the conformation of S proteins. Our previous study showed that the neutralizing antibody 55A8 is an RBD-specific antibody (22), and the results of this study demonstrated that 55A8 mediated the enhancement of SARS-CoV-2 pseudovirus by FcγRII expression in leukocyte lines, which was consistent with the mechanism of the SARS-CoV-2 monoclonal antibody 7F3 (36). Most importantly, our results also confirmed that the introduction of LALA mutations at the Fc end of 55A8 eliminated the ADE effects, indicating that 55A8 may be used as a clinical drug to prevent SARS-CoV-2 variants.

Interestingly, we noted that the variable genes of 55A8 were transcribed from IGHV1-69 and IGKV1-5, while the variable genes of the non-ADE antibodies in this study tended to be distributed in other gene clusters (Supplementary Table 2). According to the data analysis from a previous report, these two variant regions were also genetically responsible for a panel of ADE Abs (23, 24). However, this observed phenomenon with the IGHV1-69 and IGKV1-5 germline genes in ADE Abs could not be demonstrated due to insufficient data; in other words, its correlation with ADE activities remains unknown.

Additionally, another study from our team confirmed that the 55A8 reduced Omicron viral replication and prevented disease symptoms without causing additional distress in hamsters (38), indicating that the 55A8 antibody could not mediate the ADE effect of the Omicron variant *in vivo*, which was consistent with the results using the pseudoviruses system *in vitro* (Supplementary Figure 7). Since antibody-enhancing infection *in vitro* does not necessarily herald enhanced infection *in vivo*, one additional improvement that may be integrated into our study is to provide more validation experiments *in vivo* using authentic SARS-CoV-2 variants. Taken together, although rare enhanced infection was observed in the neutralizing antibodies and plasma samples, it is difficult to predict whether this phenomenon will occur in the setting of human infection or vaccination. If the rarely enhanced immunopathology was observed *in vivo*, it will be important to continue to monitor ongoing COVID-19 vaccination and neutralizing antibody drugs.

In conclusion, the rare plasma and neutralizing antibodies from convalescent patients mediated the ADE of SARS-CoV-2 variants infection *in vitro*. Thus, ADE may occur in a minority of people who have ADE antibodies and are then exposed to a newly emerged SARS-CoV-2 variant. These data suggested the ongoing neutralizing antibody drugs from convalescent patients who may need to undergo an ADE screening test by SARS-CoV-2 variants, which may benefit the safety of antibody-based therapies in future. Additionally, the potent neutralizing antibody 55A8 mediated the ADE effects depending on FcγRII, and the ADE effects of this antibody could be eliminated after Fc segment modification, indicating that this neutralizing antibody could still be used to prevent SARS-CoV-2 variants. This work also provides a reference for the development of approaches for the treatment of COVID-19 based on potential neutralizing antibodies.

## Data availability statement

The original contributions presented in the study are included in the article/Supplementary material, further inquiries can be directed to the corresponding author/s.

## Ethics statement

The studies involving human participants were reviewed and approved by the application of antibody tests patients infected with SARS-CoV-2. The patients/participants provided their written informed consent to participate in this study.

## Author contributions

AJ and YinW conceived and designed the study. YH, RW, and YinW constructed the plasmids of SARS-CoV-2 variants. FL and JH were responsible for antibody expression and purification. SS, YH, SM, and YiW performed SARS-CoV-2 variant ADE assays. SM, SS, YinW, XH, TL, CH, SL, MS, WW, and AJ generated figures and tables and take responsibility for the integrity and accuracy of data presentation. YinW, WW, and AJ wrote the manuscript. All authors contributed to the article and approved the submitted version.

## Funding

This work received financial support from the Chongqing Medical University fund (X4457). This research was also supported by the Chongqing Municipal Natural Science Foundation (cstc2018jcyjAX0194), and the Postdoctoral Science Foundation of Chongqing Municipal Natural Science Foundation (cstc2020jcyj-bshX0020).

## Conflict of interest

The authors declare that the research was conducted in the absence of any commercial or financial relationships that could be construed as a potential conflict of interest.

## Publisher's note

All claims expressed in this article are solely those of the authors and do not necessarily represent those of their affiliated organizations, or those of the publisher, the editors and the reviewers. Any product that may be evaluated in this article, or claim that may be made by its manufacturer, is not guaranteed or endorsed by the publisher.
