# Supplementary material for: Neutralizing antibodies from the rare convalescent donors elicited antibody-dependent enhancement of SARS-CoV-2 variants infection
Source: Front Med (Lausanne). 2022 Oct 19;9:952697. doi: 10.3389/fmed.2022.952697 (PMC9627283; doi:10.3389/fmed.2022.952697)
Supplement: Supplementary file 7 [file Data_Sheet_1.pdf]

Supplementary Figures:

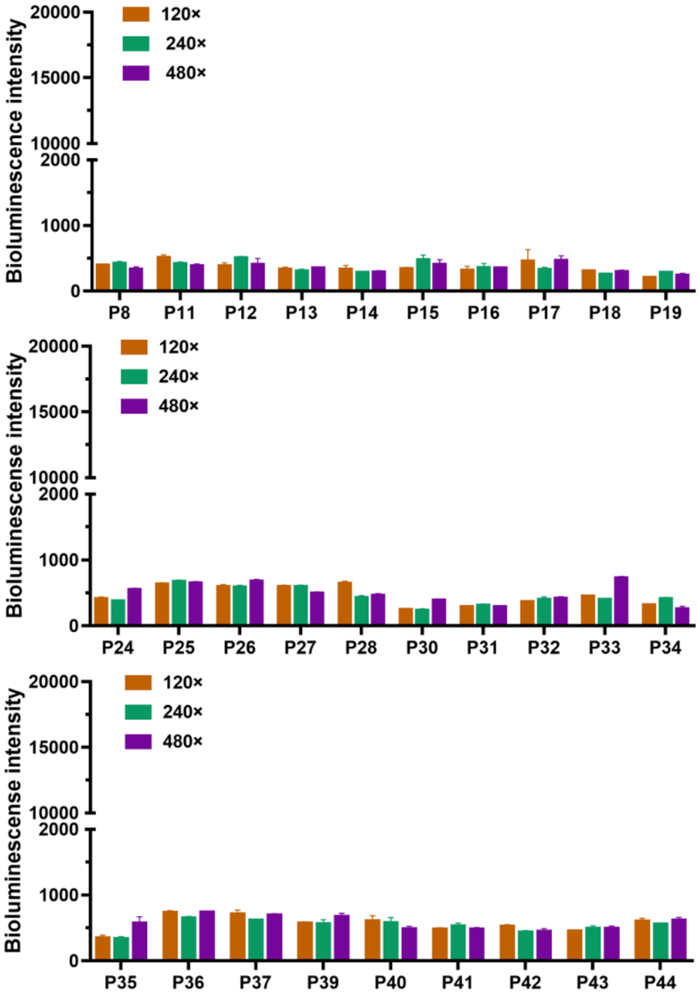

Supplementary Figure 1. ADE activities of the plasma samples from COVID-19 convalescent patients against the SARS-CoV-2 WT. The SARS-CoV-2 WT pseudovirus were pre-incubated with 29 plasma samples diluted 120, 240 and 480-fold, and these mixtures were added to Daudi cells to evaluate their ability to enhance infection. RLU values resulting from infection with variant pseudotyped viruses were quantified by luminescence meter. Data for each plasma sample were obtained from a representative infectivity experiment of three replicates, presented as the mean values ± SEM.

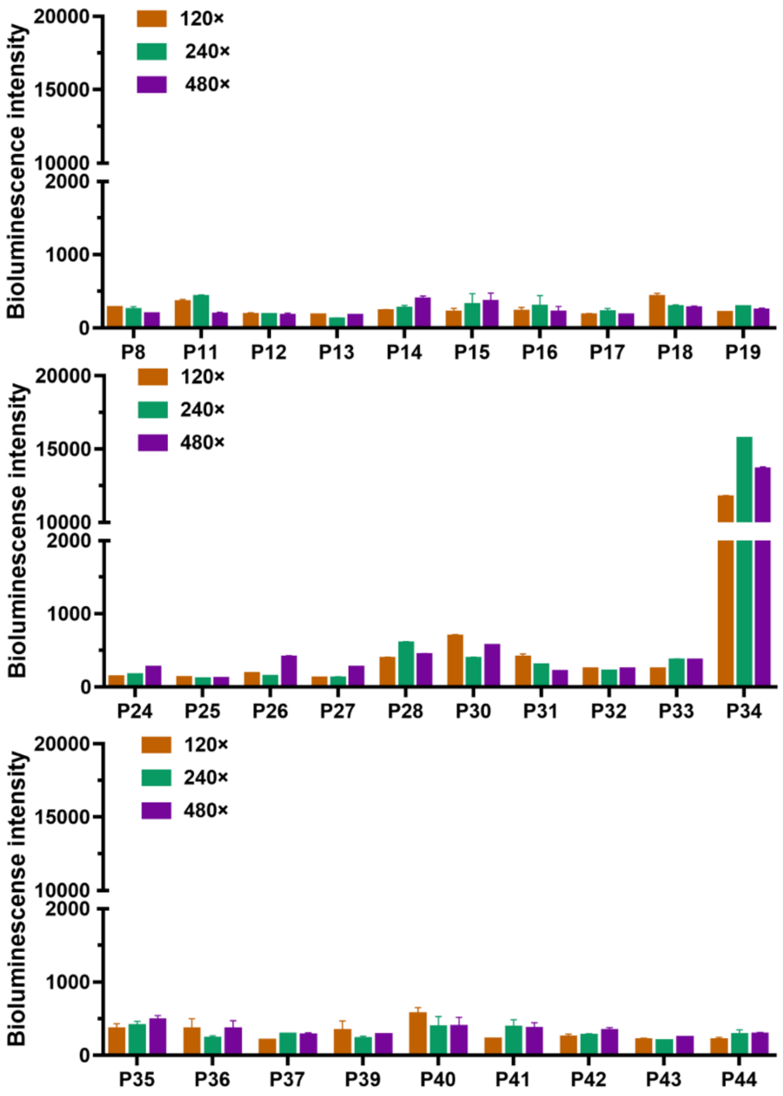

Supplementary Figure 2. ADE activities of the plasma samples from COVID-19 convalescent patients against the B.1.1.7 variant. The earliest epidemic B.1.1.7 pseudovirus were pre-incubated with 29 plasma samples diluted 120, 240 and 480-fold, and these mixtures were added to Daudi cells to evaluate their ability to enhance infection. RLU values resulting from infection with variant pseudotyped viruses were quantified by luminescence meter. Data for each plasma sample were obtained from a representative infectivity experiment of three replicates, presented as the mean values ± SEM.

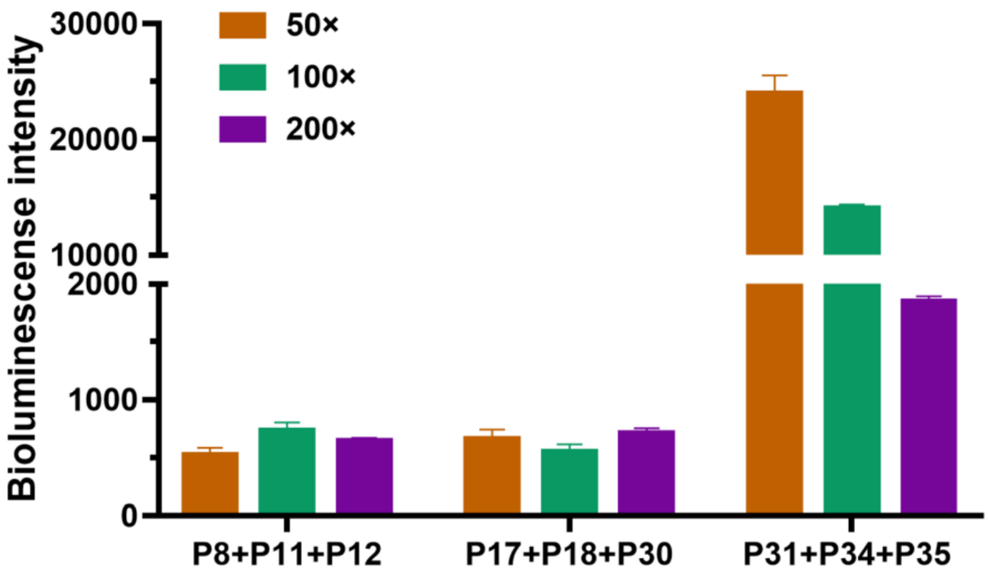

Supplementary Figure 3. ADE activities of the mixed 3 plasma samples against the B.1.1.7 variant. Since the neutralizing antibody 55A8 gene was isolated from a mixed memory B-cell sample containing the P8, P11, P12, P17, P18, P30, P31, P34 and P35 samples, this study also detected enhancement of the ADE effects for these 9 plasma samples. The epidemic B.1.1.7 pseudoviruses were pre-incubated with mixed 3 plasma samples diluted 50, 100 and 200-fold, and these mixtures were added to Daudi cells to evaluate their ability to enhance infection. RLU values resulting from infection with variant pseudotyped viruses were quantified by luminescence meter. Data for each plasma sample were obtained from a representative infectivity experiment of three replicates, presented as the mean values ± SEM.

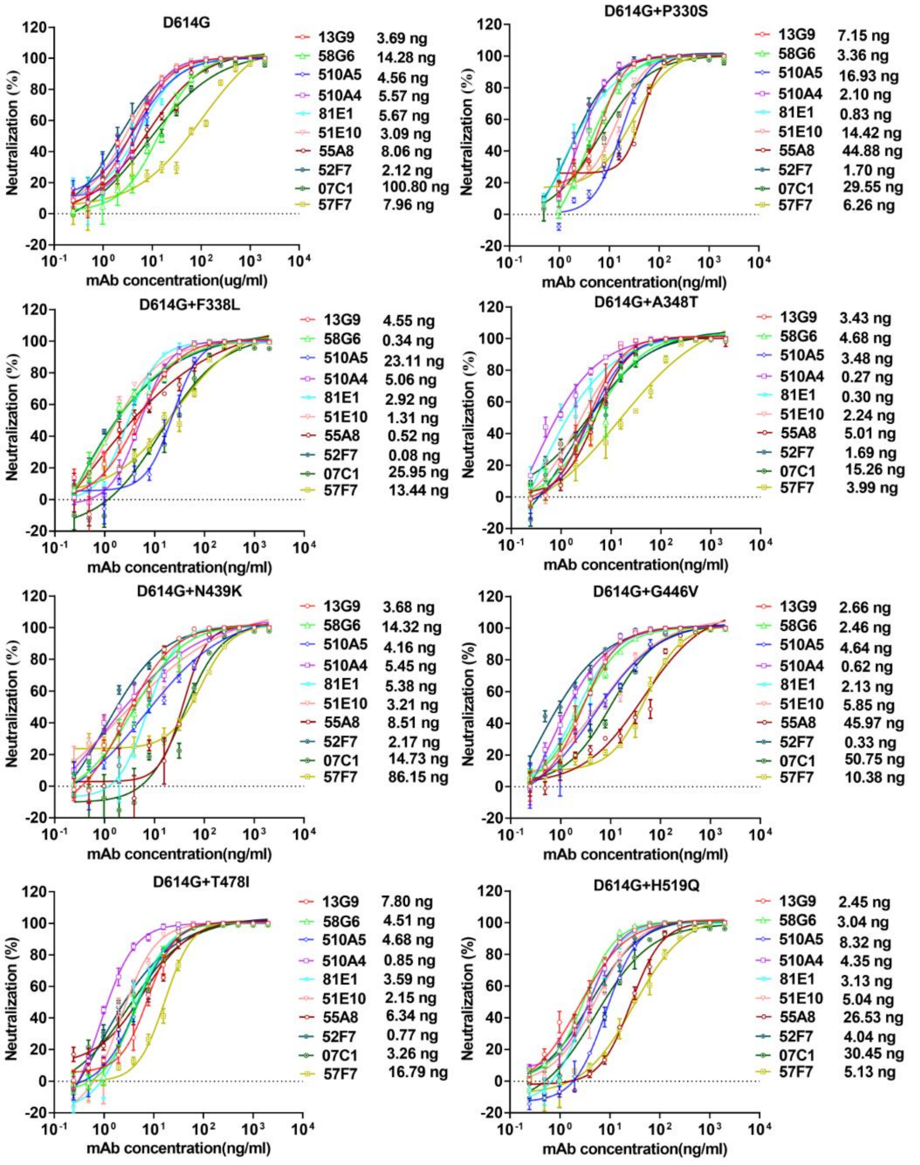

Supplementary Figure 4. The neutralizing capabilities of the neutralizing antibodies against the SARS-CoV-2 variants. The neutralizing capabilities of 10 ultrapotent Nabs (including 13G9, 58G6, 510A4, 510A5, 81E1, 51E10, 55A8, 52F7, 57F7 and 07C1) against the SARS-CoV-2 variants (including D614G, D614G+P330S, D614G+F338L, D614G+A348T, D614G+N439K, D614G+T478I and D614G+H519QSARS-CoV-2 D614G+G446V) were evaluated. IC50 were used to analyze the neutralizing activity and determined by the luminescence assay. Dashed lines indicated the rate without neutralization. Data for each NAb were obtained from a representative neutralization or binding experiment, with three replicates. Data are presented as the mean values ± SEM.

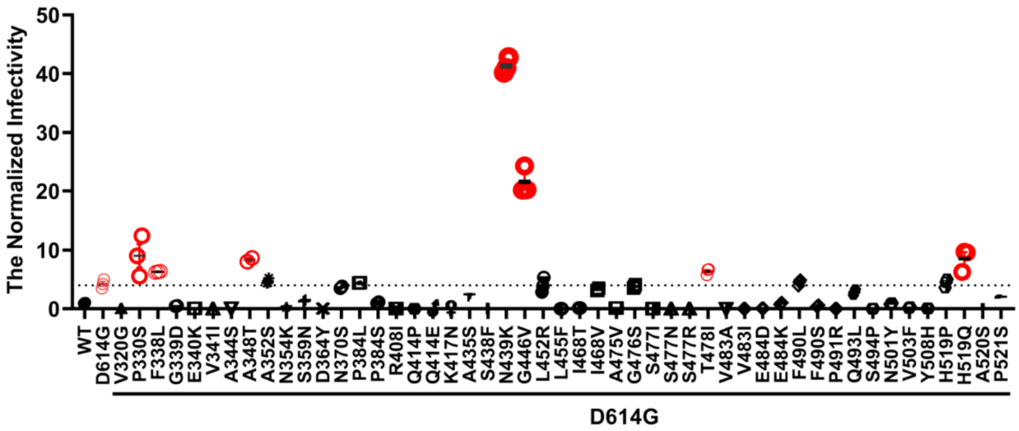

Supplementary Figure 5. Assessment of the infectivity of the engineered double-mutant variants. RLU values resulting from infection with variant pseudotyped viruses were quantified by luminescence meter and normalized to the reference strain (Wuhan-1) produced in parallel, with the difference by 4-fold being considered as significant. The horizontal dashed lines indicate the threshold of 4-fold difference. Data for each variant were obtained from a representative infectivity experiment of three replicates, presented as the mean values ± SEM.

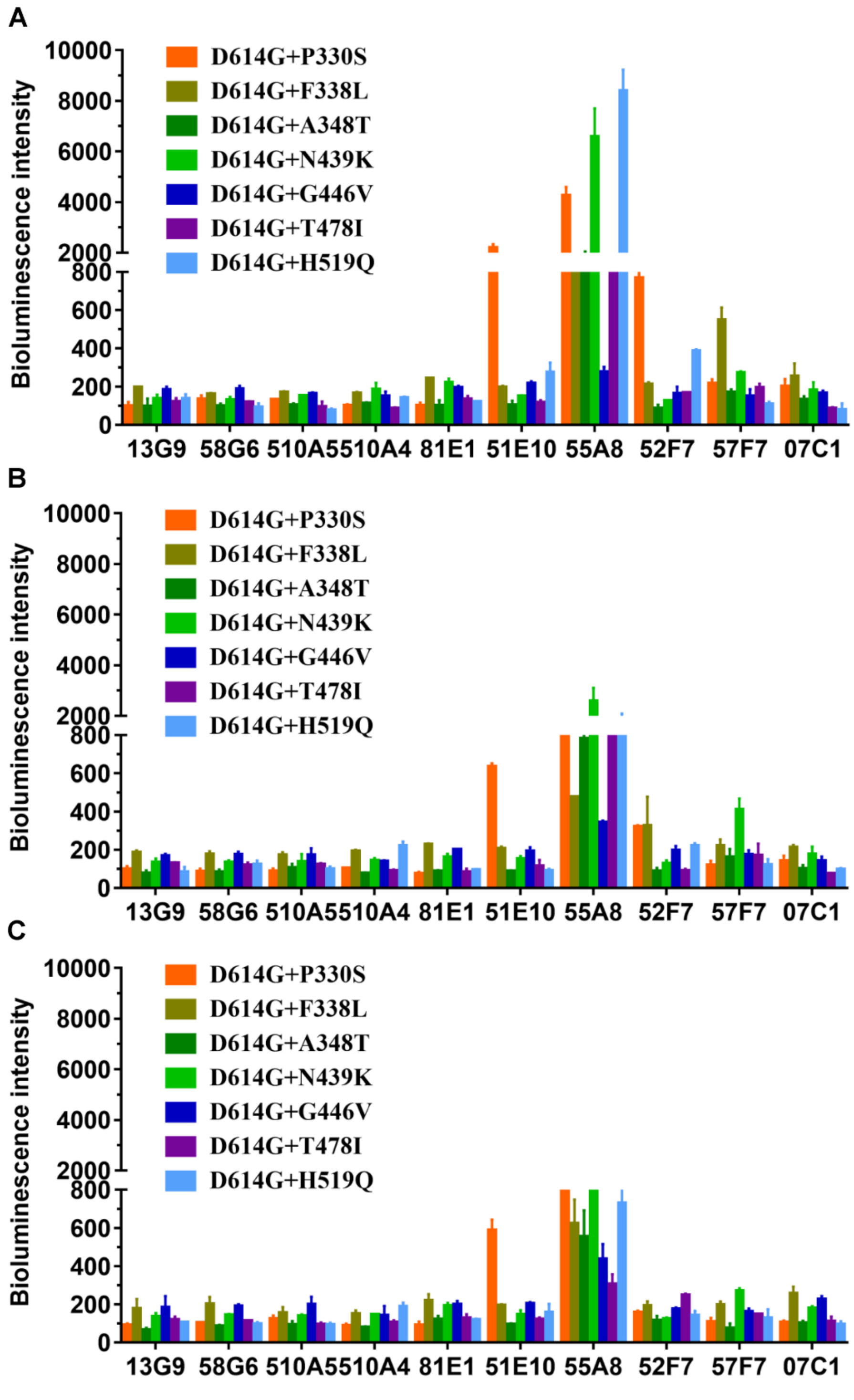

Supplementary Figure 6. The ADE activities of the top 10 neutralizing antibodies (NAbs) for the high infective double-mutant variants. Assessment of the ADE activities of 10 ultrapotent NAbs (including 13G9, 58G6, 510A4, 510A5, 81E1, 51E10, 55A8, 52F7, 57F7 and 07C1) for the SARS-CoV-2 double-mutant pseudovirus. Pseudoviruses pre-incubated with (A) 250, (B) 500 and (C) 1000 ng/ml NAbs mixtures were added to Daudi cells to evaluate their ability to enhance infection.

RLU values resulting from infection with variant pseudotyped viruses were quantified by luminescence meter. Data for each NAb were obtained from a representative infectivity experiment of three replicates, presented as the mean values ± SEM.

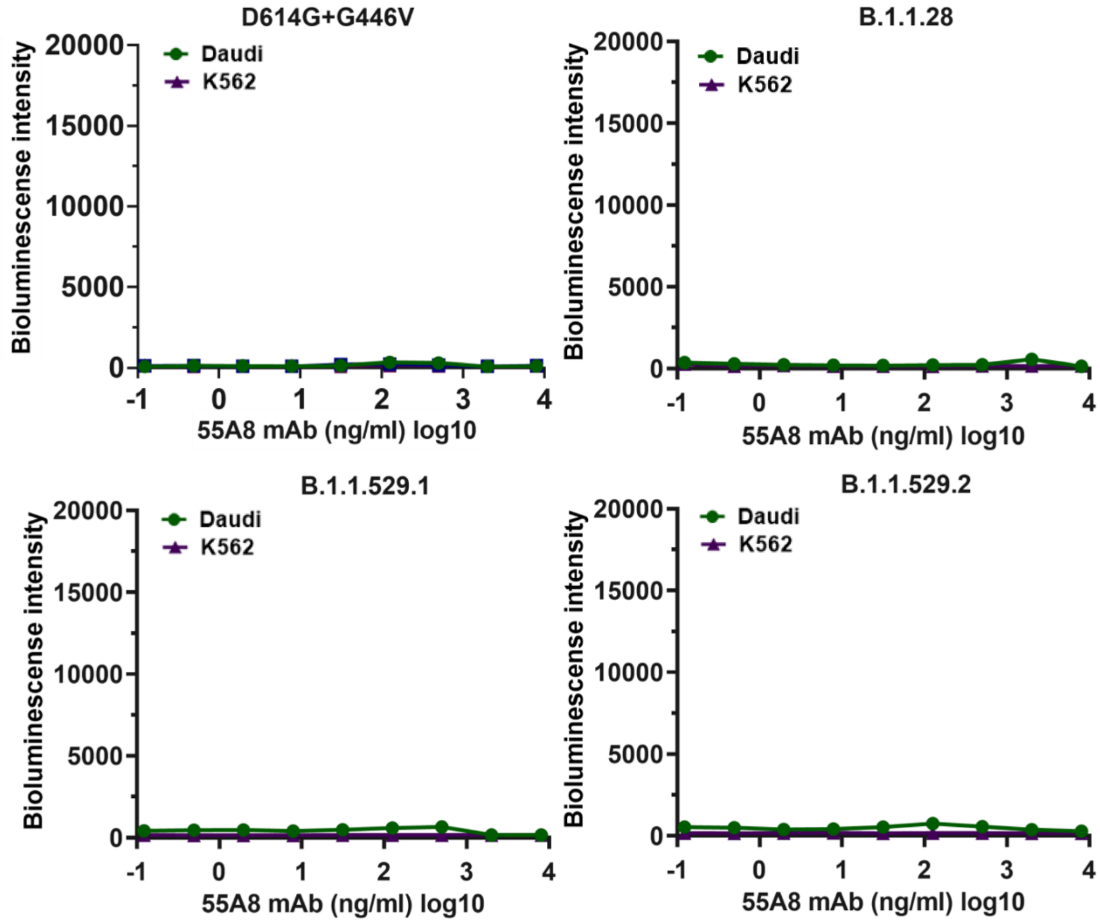

Supplementary Figure 7. The ADE activities of the neutralizing antibody 55A8 for the D614G+G446V, B.1.1.28, B.1.1.529.1 and B.1.1.529.2. Pseudoviruses pre-incubated with serial dilutions of 55A8 mixtures were added to Daudi, Raji and K562 cells to evaluate their ability to enhance infections. RLU values resulting from infection with variant pseudotyped viruses were quantified by luminescence meter. Data for each NAb were obtained from a representative infectivity experiment of three replicates, presented as the mean values ± SEM.

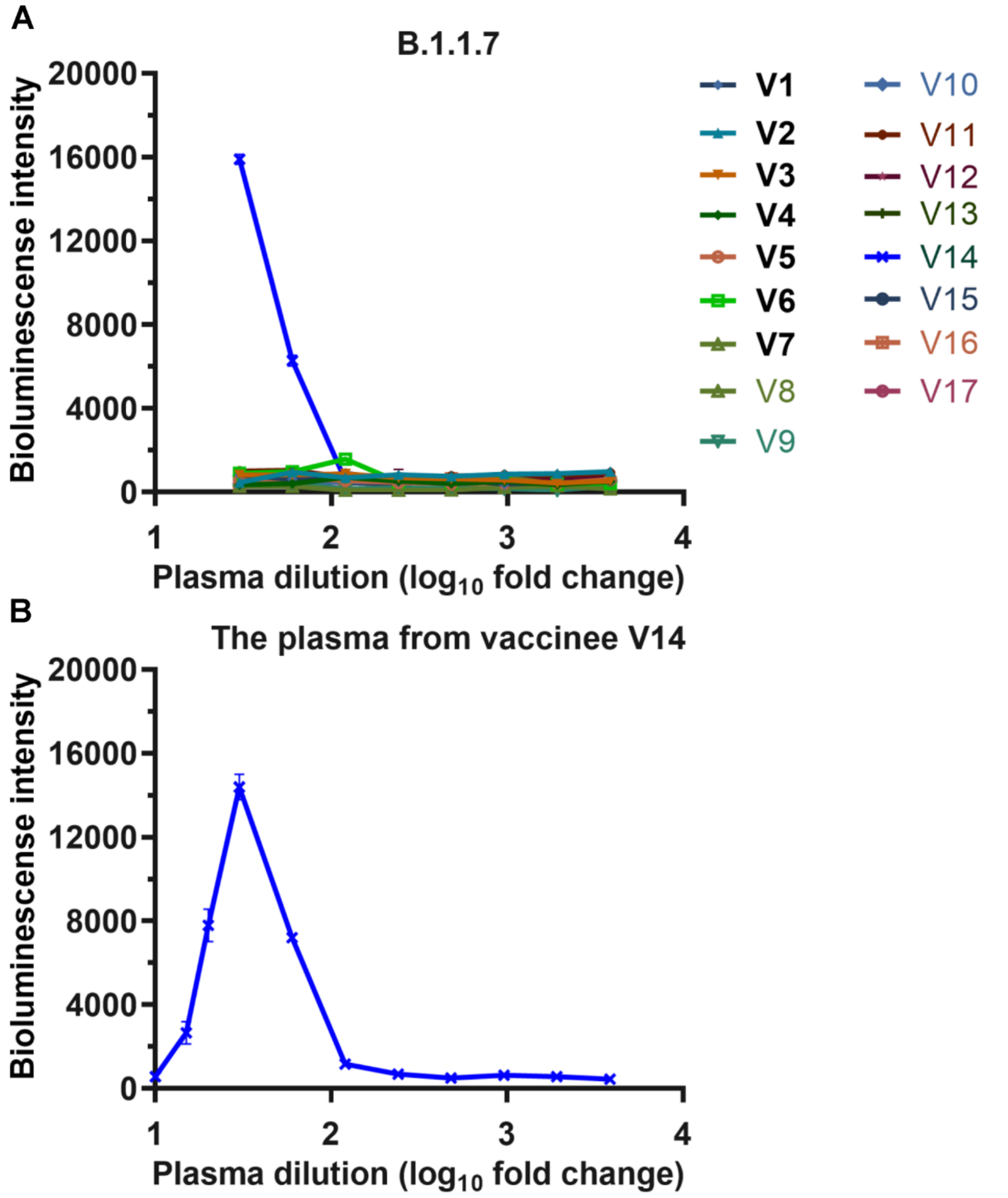

Supplementary Figure 8. ADE activities of the plasma samples from the SARS-CoV-2 **vaccinees. (A)** The enhancement of B.1.1.7 infection for the 17 vaccinees' plasma samples**.** The B.1.1.7 pseudoviruses were pre-incubated with serially dilution 17 vaccinees' plasma samples (diluted 30, 60, 120 ,240, 480, 960, 1920 and 3840-fold), and the mixtures were added to Daudi cells to evaluate their ability to enhance infection. Each curve represents an individual plasma sample. RLU values resulting from infection with variant pseudotyped viruses were quantified by a luminescence meter. Data for each plasma sample were obtained from a representative infectivity experiment of three replicates and

are presented as the mean values ± SEM. **(B)** The enhancement of B.1.1.7 infection for the V14 plasma sample. The B.1.1.7 pseudoviruses were pre-incubated with serially dilution V14 plasma samples (diluted 10, 15, 20, 30, 60, 120 ,240, 480, 960, 1920 and 3840-fold), and other conditions are the same as (A).
